# Local, systemic and immunologic safety comparison between xenogeneic equine umbilical cord mesenchymal stem cells, allogeneic canine adipose mesenchymal stem cells and placebo: a randomized controlled trial

**DOI:** 10.3389/fvets.2023.1098029

**Published:** 2023-05-17

**Authors:** Eva Punzón, María García-Castillo, Miguel A. Rico, Laura Padilla, Almudena Pradera

**Affiliations:** ^1^EquiCord S. L., Alcorcón, Madrid, Spain; ^2^LEITAT Technological Center, Barcelona, Spain

**Keywords:** xenogeneic MSC transplantation, umbilical cord mesenchymal stem cell, xenogeneic, safety, intra-articular administration, dogs, mesenchymal stem cell, DogStem

## Abstract

Mesenchymal stem cells are multipotent cells with a wide range of therapeutic applications, including, among others, tissue regeneration. This work aims to test the safety (EUC-MSC) of intra-articular administration of equine umbilical cord mesenchymal stem cells in young healthy dogs under field conditions following single and repeated administration. This was compared with the safety profile of allogenic canine adipose derived mesenchymal stem cells (CAD-MSC) and placebo in order to define the safety of xenogeneic use of mesenchymal stem cells when administered intra-articular. Twenty-four police working dogs were randomized in three groups in a proportion 1:1:1. EUC-MSCs and CAD-MSCs were obtained from healthy donors and were manufactured following company SOPs and under GMP and GMP-like conditions, respectively, and compliant all necessary controls to ensure the quality of the treatment. The safety of the treatment was evaluated locally, systemically and immunologically. For this purpose, an orthopedic examination and Glasgow test for the assessment of pain in the infiltrated joint, blood tests, clinical examination and analysis of the humoral and cellular response to treatment were performed. No adverse events were detected following single and repeated MSC administration despite both equine and canine MSC generate antibody titres in the dogs. The intra-articular administration of equine umbilical cord mesenchymal stem cells in dogs has demonstrated to be safe.

## 1. Introduction

Mesenchymal stem cells (MSCs) are multipotent cells that can be isolated from many different adult and neonatal tissues. They are defined by their phenotype and functional properties, such as: spindle-shaped morphology, adherence to plastic, immune response modulation capacity, and multilineage differentiation potential (1). Accordingly, MSCs have a wide range of promising applications in the treatment of inflammatory diseases, autoimmune disorders, tissue repair and regeneration (2).

Due to their immunomodulatory properties (3) MSCs are being widely investigated in veterinary medicine for the treatment of inflammatory and autoimmune diseases (4). Also, it is known that through their immunomodulatory capacity they possess tissue regenerative properties (5–7), which is why they are also being investigated for their use in wound healing, tendinopathies, nerve injuries, etc. (4).

Among the pathologies for which MSCs are attracting interest is osteoarthritis (OA). Since current OA treatments treat the symptoms but not the underlying pathology (therefore cartilage deterioration continues) biological therapies based on MSCs have become of great interest in both human and veterinary osteoarthritis research (8). MSCs have demonstrated to be able to reduce local and systemic inflammation due to their immunomodulatory capabilities. In addition, MSCs seem to contribute to cartilage healing through their paracrine signalling which stimulate local repair (9–11).

To date, it has been widely considered that MSCs were immune-privileged, even between different species (12–14). However, recently it has been shown that after single and repeated allogeneic or xenogeneic MSC application the recipient may develop mild antibody titters to donor cells (15). This finding would suggest MSCs are immune-tolerant rather than immune-privileged (16, 17). Despite the induction of serologic response, it has not been involved in any case with systemic immunologic reactions following the application of allogeneic or xenogeneic cells. The lack of association between the presence of antibodies and the onset of clinical symptoms may be due to antibodies generated in the recipient were at very low titters, thus insufficient to provoke a clinically relevant immune response (15, 16). This may allow a patient to receive MSCs from different sources: the patient can receive his own MSCs (autologous MSCs), MSCs from a donor (allogeneic MSCs) or MSCs from a donor of a different species (xenogeneic MSCs) (8).

The intra-articular safety of autologous (18, 19) and allogeneic (20) canine MSCs is well reported in the literature.

The treatment with autologous MSCs has the advantage of not involving donor animal harvesting, so it does not imply ethical issues (21). However, it is a time-consuming task that requires a surgical procedure to harvest tissue containing MSCs (e.g., adipose, bone marrow) from the animal, which is used to grow MSCs. Autologous treatments have a big variability in terms of viability, population doublings and other manufacturing characteristics that have impact in the efficacy of the product and this can be influenced by the manufacturing process and the donor themselves, as the quality of the MSCs declines with increasing donor age (22). Allogeneic and xenogeneic MSC offers the possibility of producing homogenous off-the-shelf treatments so there is not waiting time for growing the MSCs and the animal do not need to have surgery in order to remove its own tissue (23).

Allogeneic cells might be thought to have advantages over xenogeneic cells as they are expected to have higher donor-host compatibility (23). However, there is evidence pointing at xenogeneic MSCs having a comparable effectiveness and safety profile to allogeneic MSCs (24–26), with the additional advantage, among others, of being absent of species-specific transferable pathogens (8). Another advantage of xenogeneic stem cells is the utilisation of a donor species with a higher culture capacity than the recipient (10).

For these reasons, the use of xenogeneic MSCs has been explored by some authors, demonstrating their safety and efficacy after single and repeated administrations (15, 27). Moreover, our group has demonstrated the safety and efficacy of EUC-MSC in natural occurring canine OA (28).

In this work the treated species is the dog and the tissue of choice for the extraction and culture of MSCs is the equine umbilical cord (EUC).

Umbilical cord (UC) presents important advantages as source of MSCs: non-invasive sourcing, higher proliferation capacity (29), greater immune-modulatory capacity (30), less immunogenic (31) and a more secure profile with less risks derived from possible cell mutations, viral agents, parasites agents or other contaminants (32).

Allogeneic MSCs from canine UC are not easy to obtain. It is almost impossible to obtain the canine UC as the dam’s ingests the placenta and UC after birth, therefore the only way to obtain the tissue would be after C-section surgery. Conducting a surgical procedure or C-section in order to obtain UC for pharmaceutical development purposes gives rise to the question of legitimacy and is definitely not aligned with animal welfare. For these reasons, the allogeneic use of canine MSCs from UC is not a good or viable option (33). In addition to being difficult and expensive to obtain (surgery or any other complex process), due to the small size of the tissue, cells require more expansion, passes and population doublings, which are known to have negative effects in terms of efficacy and safety of the product (34).

EUC provides a good alternative, because it is an easy tissue to obtain, virtually limitless that is discarded after birth and the mare does not instinctively ingest it. This makes it perfect from an ethical and animal welfare point of view. In addition, it is a large tissue (~1 kg), rich in MSCs (172,000 cell/g), therefore a large number of cells, requiring minor cell expansion, can be obtained (35).

The aim of this study is to demonstrate that the xenogeneic use of MSC is as safe as the allogeneic use. To this end the safety of intra-articular administration of EUC-MSC in young healthy dogs under field conditions in single and repeated administration was compared with the allogeneic use of canine adipose mesenchymal stem cells (CAD-MSCs) and placebo in order to define their safety profile in the treatment of osteoarthritis.

The study had a total duration of 9 weeks where patients were monitored for clinical signs on a regular basis, as well as orthopaedic signs of the infiltrated joint. Antibodies generated by the dogs against EUC-MSCs, CAD-MSCs and Placebo have also been monitored regularly. Finally, a possible cellular memory response (mediated by CD8 lymphocytes) has been studied, which, if present, could generate an exacerbated immune response after re-infiltration.

## 2. Materials and methods

### 2.1. Design

The present study is a double-blinded (owner and researcher), parallel group, randomized and placebo- controlled trial. It was carried out following the International Cooperation on Harmonisation of Technical Requirements for Registration of Veterinary Medicinal Products for Good Clinical Practice (VICH guidelines) and satisfied national regulatory and animal welfare standards and requirements. Informed consent was obtained from dog owner prior to inclusion.

The study was conducted according to the schedule on Table 1.

**Table 1:** Study schedule.

Activity	First admin (day 0)	Daily visits (day 1–6)	Day 7	Day 14	Day 21	Second admin (day 28)	Daily visits (day 29–34)	Day 35	Day 42	Day 49	Day 56	Day 63
Biochemistry and hematology	x					x					x	
Intra-articular administration	x					x						
Clinical exam	x	x	x	x	x	x	x	x	x	x	x	x
Orthopedic exploration of the treated joint	x	x	x	x	x	x	x	x	x	x	x	x
Glasgow scale determination	x	x	x	x	x	x	x	x	x	x	x	x
Humoral response	x								x			
Cellular response	x								x			
Registration adverse events		x	x	x	x	x	x	x	x	x	x	x
Registration concomitant drugs	x	x	x	x	x	x	x	x	x	x	x	x
Study completion												x

On day 0 and day 28 (administration days), before product administration, a blood sample was extracted for haematology and biochemistry. On these visits it was also performed a clinical exploration and an orthopaedic evaluation of the joint to ensure that it was not affected prior to infiltration.

After this, all the animals received one intra-articular dose of EUC-MSCs, CAD-MSC or placebo in the right knee.

As disclosed in Table 1, for the first 6 days after the two administrations, the dogs received daily safety visits and weekly visits on days 7, 14, 21, 28, 35, 42, 49, 56 and 63 with a margin of ±2 days for the weekly review. Caregivers were trained to detect any potential adverse effect (AE) and dogs were monitored daily by them. The expected AE were local heat, inflammation and lameness after administration.

Each visit was conducted according to the schedule on Table 1. Any abnormal health observation irrespective of their nature and severity made by either owner or veterinarian was recorded according to VICH guidelines.

### 2.2. Animal selection

The animals were active police dogs belonging to the *Centro Cinológico de la Guardia Civil*. It was a uniform population in terms of size, feeding and lifestyle. They were German Shepherds, Malinois and Labrador dogs.

The inclusion criteria were: healthy young animals (older than 1 year), between 20 and 40 kg body weight, with normal haematology and biochemistry. Dogs showed no orthopaedic discomfort at exploration.

Table 2 describes the main characteristics of the population.

**Table 2:** Characteristics of the study population.

ID	Breed	Sex	Birth date	Group
1	Labrador	Male	12/08/2016	Placebo
2	Labrador	Male	21/11/2015	EUC-MSCs
3	Labrador	Male	11/12/2016	CAD-MSCs
4	Labrador	Male	04/04/2016	CAD-MSCs
5	Leonese sheepdog	Male	13/12/2016	EUC-MSCs
6	German shepherd	Male	20/12/2016	EUC-MSCs
7	Labrador	Male	30/07/2016	EUC-MSCs
8	German shepherd	Male	17/03/2016	Placebo
9	Labrador	Male	30/07/2016	CAD-MSCs
10	Labrador	Male	01/10/2016	Placebo
11	Malinois	Male	21/12/2016	CAD-MSCs
12	Malinois	Male	27/07/2016	Placebo
13	German shepherd	Male	20/06/2016	EUC-MSCs
14	Malinois	Female	06/10/2016	CAD-MSCs
15	Malinois	Female	17/02/2014	EUC-MSCs
16	German shepherd	Female	14/11/2016	EUC-MSCs
17	Malinois	Female	27/07/2016	Placebo
18	Labrador	Female	21/11/2015	Placebo
19	Labrador	Female	12/01/2016	EUC-MSCs
20	Labrador	Female	12/01/2016	Placebo
21	Malinois	Female	08/03/2016	CAD-MSCs
22	German shepherd	Female	01/06/2015	Placebo
23	Golden retriever	Female	10/04/2014	CAD-MSCs
24	Malinois	Female	20/09/2015	CAD-MSCs

The age of each dog should be calculated taking into account that the study was carried out by February 2018.

### 2.3. Treatments

Twenty-four dogs that met the inclusion criteria were randomized in three groups of treatment (1:1:1) with: equine cells EUC-MSCs (8), canine cells CAD-MSCs (8) or placebo (8).

Dogs in the EUC-MSC group received 7.5 × 10^6^ EUC-MSCs (DogStem®) intra-articular. Dogs in the CAD-MSC group received 7.5 × 10^6^ CAD-MSCs, at day 0 and 28 of the study.

Both MSCs types were thawed and placed in culture for recovery. They were then harvested and packed in 1 ml of vehicle consisting on a DMSO-free and protein-free solution of salts, sugars and antioxidants. Dogs in the placebo group received 1 ml of saline.

### 2.4. MSCs manufacturing

For this work, EUC from one donor was collected from a concerted stud after the natural birth of a male foal and it was processed. EUC-MSCs were isolated from Wharton’s jelly, expanded in primary culture until passage 4 following company SOPs, European regulations and under Good Manufacturing Practices (GMP) conditions. Cells were passed when they reached 90% confluence using trypsin 1X (Life Thecnologies).

For the obtaining of CAD-MSCs, the adipose tissue sample was obtained during a surgical intervention for castration from a healthy young female donor. Cells were expanded in primary culture under GMP-like conditions up to passage 4.

Both tissues underwent enzymatic digestion with collagenase type I (Gibco) 1 mg/ml for 3 h for adipose tissue and 4 for UC MSCs were cultured in Dulbecco’s modified Eagle’s medium (DMEM; Gibco) with 10% foetal bovine serum (FBS; Gibco) and 1% Penicillin/streptomycin (Gibco).

After the expansion of the cells of both final products (EUC-MSCs and CAD-MSCs), consisting of 7.5 × 10^6^ MSCs per vial, were tested for sterility, cell concentration, viability, morphology, accumulative population doublings, mycoplasma contamination and were characterised by flow cytometry following Eu.Ph 2.6.27 for sterility, Eu.Ph 2.7.29 for cell concentration and viability Eu.Ph 2.6.7 for mycoplasma and Eu. Ph 2.7.24 for flow cytometry. For morphology and accumulative population doublings a justification and validation of the method was provided to the EMA for approval. For characterisation MSCs must be positive for CD90 (BD Biosciences; 5E10) and CD44 (Bio-Rad; CVS18 for equine; R&D Systems; 69-S5 for canine) and negative for MHC-II (Bio-Rad; CVS20) and CD45 (Bio-Rad; F10-89-4 for equine and Bio-Rad; YKIX716.13 for canine) by using the CytoFLEX flow cytometer and CytExpert 2.0 Software for data analysis.

In addition, a potency test was performed consisting on the ability of MSCs to inherently secrete prostaglandin E-2 (R&D Systems Parameter Prostaglandin E2), as it has been shown that the immunomodulatory capacity of the cells depends on their ability to secrete this molecule (36). The specification for a batch of MSCs to be compliant is [PGE2] ≥2,366 pg./ml.

### 2.5. Orthopaedic exploration

Orthopaedic exploration was performed by the assessment of joint effusion, detection by palpation of patellar ligament through medial approach, lameness at walk, joint palpation and inspection of injection point. The same veterinary surgeon made the orthopaedic exploration for every dog and at all the time points. For the evaluation of joint effusion, lameness at walk, joint palpation and inspection of injection point the criteria was “yes/no.” A minimum of lameness would be considered lameness and the same for any sign of discomfort of the animal to manipulation of the joint: it would be considered pain.

On day 0 and 28 (before product administration) a drawer test was performed in order to discard rupture of cruciate ligament.

The orthopaedic exploration was assessed on every visit performed.

### 2.6. Clinical examination

The parameters assessed included: overall status (including hydration, lymph nodes and abdominal palpation) mucous membranes colour, pulmonary auscultation and breathing frequency, cardiac auscultation, heart rate, and rectal temperature. The same veterinary surgeon made the clinical examination for every dog and at all the time points.

The clinical examination was performed on every scheduled visit.

### 2.7. Glasgow scale determination

Glasgow Composite Measure Pain Scale short form (CMPS-SF) was developed by Glasgow University to measure acute pain in dogs (surgical, medical, inflammatory, or traumatic) and was designed as a clinical decision-making tool. It was constructed using psychometric methods and has been shown to be valid, reliable, and responsive to clinical change in a range of clinical settings (37). The CMPS-SF is used to establish when a patient needs analgesic treatment. The recommended analgesic intervention level was 6/24. Therefore, if an animal score was 6 or above out of 24, analgesic treatment (0.2 mg/kg body weight of meloxicam) was administered.

CMPS-SF comprises six behavioural categories with associated descriptive expressions (38): vocalisation, attention to wound, mobility, response to touch, demeanour, and posture/activity. Items are placed in increasing order of pain intensity and numbered accordingly.

The Glasgow scale was assessed on every visit and by the same veterinary surgeon.

### 2.8. Laboratory examinations

Blood samples were collected when indicated in Table 1 for haematology (red blood cells, haemoglobin, haematocrit, medium corpuscular volume, whole blood cells and blood smear formula of Eosinophils, Basophils, Lymphocytes, Monocytes, and platelet count) and serum biochemistry (Blood Urea Nitrogen (BUN), Creatinine, Aspartate Aminotransferase (AST), Alanine Aminotransferase (ALT), Total Proteins, Alkaline phosphatase, Glucose, Amylase, Total bilirubin, Cholesterol, Albumin and Globulins).

Blood samples were analysed by the same external laboratory.

### 2.9. Humoral response

**Figure 1:**
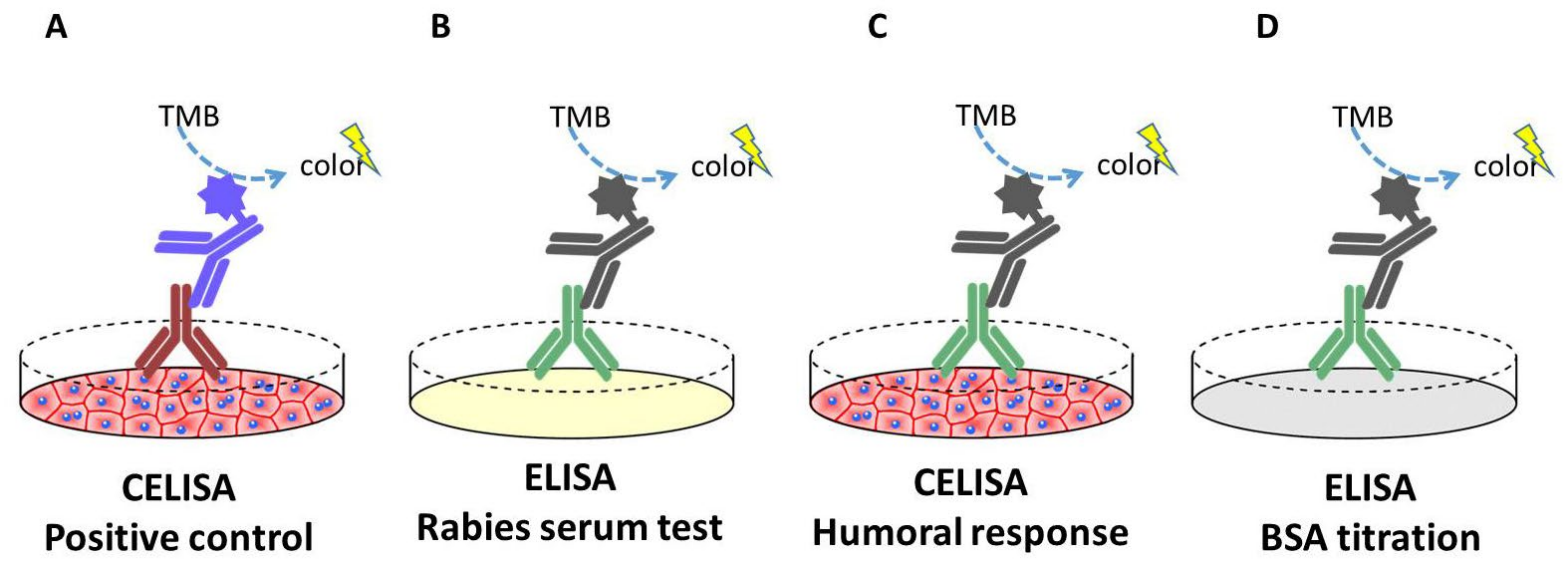
Schematic representation of the CELISA and ELISA assays performed in this study. **(A)** Positive control: CELISA where plates were coated with live EUC-MSCs or CAD-MSCs. After the incubation of mouse anti-CD44 or mouse anti-CD90 primary antibody (brown), signal was quantified. **(B)** Rabies serum test: ELISA where plates were coated with rabies vaccine antigen. After the incubation of the dog sera (green), signal was quantified. **(C)** Humoral response test: CELISA where plates were coated with live EUC-MSCs or CAD-MSCs. After the incubation of the dog sera (green), signal was quantified. **(D)** BSA titration: ELISA where plates were coated with bovine serum albumin. After the incubation of the dog sera (green), signal was quantified.

For the analysis of the humoral response against the MSCs, a CELISA (Cellular enzyme-linked immunospecific assay) was performed (Figure 1). Sera samples of all dogs were taken at day 0 and day 42. For the CELISA, EUC-MSCs or CAD-MSCs were seeded onto cell culture treated flat bottom 96-well microplates (Nunc) at a density of 20,000 cells per well and 200 μl/well of Dulbecco’s Modified Eagle Medium (DMEM)-10% foetal bovine serum (FBS) and let to adhere at 37°C and 5% CO_2_ for 24 h. The day after, supernatant was removed, and primary antibodies (positive control) or sera from placebo, CAD-MSC and EUC-MSC treated dogs (test sample) were added diluted in 100 μl/well of DMEM-10% FBS and incubated for 90 min at 37°C and 5% CO_2_. Sera from placebo dogs were exposed to both CAD-MSCs and EUC-MSCs. As positive control, mouse monoclonal anti-equine CD44 (Clone 69-S5, R&D Systems) and mouse monoclonal anti-equine CD90 (Clone MRC OX-7, Abcam) were used at 2 μg/ml. Reactivity of control antibodies against equine antigens was predicted bibliographically (39, 40) and confirmed experimentally using several concentrations of antibody on equine MSCs and control cells (negative for CD44 and CD90) (data not shown). After the incubation, wells were washed with 300 μl/well of PBS. Then, the horseradish peroxidase (HRP) conjugated secondary antibody goat anti-mouse (Jackson Immunoresearch) at a dilution of 0.04 μg/ml or rabbit anti-dog (Jackson Immunoresearch) at a dilution of 0.8 μg/ml were incubated in 100 μl/well for 30 min at 37°C and 5% CO_2_. After the incubation, wells were washed 5 times with 300 μl/well of PBS. Finally, 100 μl/well of 3,3′,5,5’-Tetramethylbenzidine (TMB) substrate was added. After 15 min at room temperature (RT), reaction was stopped by adding 100 μl/well of 1 M HCl, and signal was read at 450 nm using a Thermo Labsystems Multiskan Ascent plate reader. Background correction (OD450nm 0.3UA) was not applied. All conditions were represented in duplicates and the dilution used was 1/1000.

Rabies vaccination was used as positive control. In this assay, the antigen of the rabies vaccine vial (Etadex®, Ecuphar) was diluted 1/1,000 in PBS and coated onto the ELISA plates (MaxiSorp™, Nunc). Additionally, dogs’ sera were tested in ELISA plates coated with 5 mg/ml of bovine serum albumin (BSA fraction V, from Calbiochem) at Day 0. This control is included in order to test the presence of xenogeneic antibodies in dogs’ blood. In both assays, dog sera were analysed as previously explained in the CELISA assay.

The readout of the CELISA was qualitative (presence/absence). Quantification was not performed.

### 2.10. Cellular response

The secondary cellular response is measured in terms of an increase in the percentage of CD8+ lymphocytes within the PBMC population. For this purpose, Peripheral Blood Mononuclear Cells (PBMCs) obtained from blood samples of day 42 per protocol (after the second administration) of all the EUC-MSCs treated dogs and 3 placebo dogs were co-cultured with EUC-MSCs for 4 days. CAD-MSCs group was not included since sample was not available.

Fresh blood samples from recipient dogs were used for PBMCs isolation by Ficoll® and cryopreserved in liquid nitrogen. Viability and cell number were checked by dye-exclusion method before the assay was performed.

10Gy irradiated EUC-MSCs were seeded in 96-well plates at 20,000 cells/well in 200 μl DMEM and culture at 37°C 5% CO_2_. After 48 h, EUC-MSCs monolayer was washed once with PBS and 100,000 thawed PBMCs from dogs were seeded on top in 200 μl of Roswell Park Memorial Institute (RPMI) medium. Each condition was seeded in triplicate. The co-culture was continued for 4 days at 37°C and 5% CO_2_. As positive control PBMCs were activated with 10 μg/ml phytohemagglutinin (PHA; Sigma Aldrich). As negative control PBMCs were cultivated alone (basal condition).

On the fourth day of co-culture, PBMCs of all conditions were collected in order to determine, by flow cytometry, whether the percentage of memory lymphocyte CD8+ had increased due to the exposure with MSCs. In addition, at the end of the co-culture period, MSCs were assessed for cell number and viability to rule out the possibility that the PBMCs had reacted against them.

In order to deplete the immunomodulatory capacity of MSCs so they do not interfere with the potential memory response of the PBMCs against the MSCs a control is added. Indomethacin is used as a blocker of the production of PGE2 by the MSCs (41). It is known that the mechanism of action of MSCs is through PGE2 (36, 42). In this condition, EUC-MSCs were co-cultured with pre-treated dogs’ PBMCs in presence of indomethacin 10 μM as previously described by Carrade Holt et al. in MSCs from different sources (41). According to bibliography this concentration is enough to block completely the secretion of PGE2.

**Figure 2:**
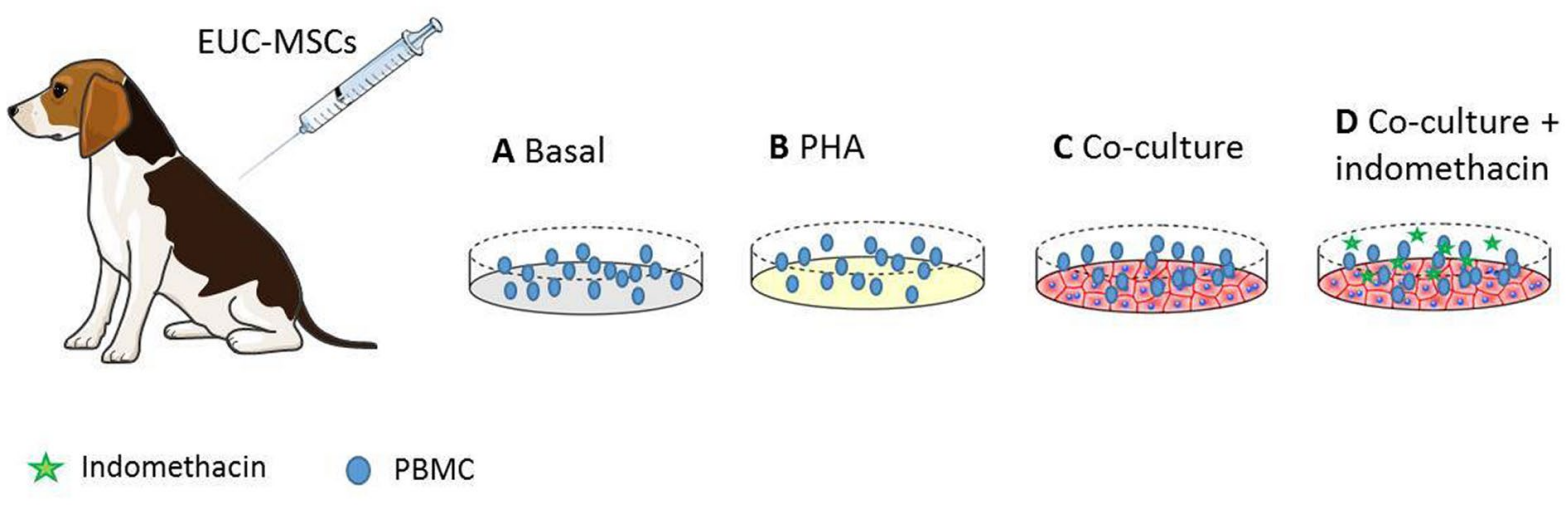
PBMCs from EUC-MSCs treated dogs were extracted and cultured in the four experimental conditions represented above. **(A)** Basal PBMCs are incubated in culture media. **(B)** Shows the positive control where PBMCs are stimulated with PHA. **(C)** The experimental condition, where the PBMCs are co-cultured with EUC-MSCs. **(D)** The control with indomethacin. In this condition the immunosupresive capacity of EUC-MSCs is blocked.

Approximately 300,000 PBMCs per condition (Figure 2) were distributed into eppendorf tubes and washed by centrifugation at 1,800 rpm for 10 min at RT. Subsequently, the supernatant was discarded by decantation and the resulting pellet was labelled with CD8-FITC monoclonal antibody (clone YCATE55.9; BioRad) specific for dog or with an FITC isotypic control Rat IgG2a antibody at a concentration of 10 μl/10^6^ cells in a final volume of 100 μl of PBS.

All cells were incubated for 20 min at 2–8°C. After incubation, about 900 μl of PBS was added to all tubes and they were washed by centrifugation at 1,800 rpm for 10 min at RT to remove excess antibody.

Subsequently, the supernatant was discarded by decantation and the resulting pellet was resuspended in 450 μl of PBS.

To discard dead cells in analysis, 2 μl of Propidium Iodide was added to each tube just prior to cell acquisition. A total of 100,000 viable cells per condition were acquired using the CytoFLEX flow cytometer and were analysed using CytExpert 2.0 Software. For the analysis of the CD8 population, 20.000 events in the lymphocytes region were gated according to their forward (FSC-H) and sideways (SSC-H) scatter. Over this population the CD8 positive cells were analysed (43).

### 2.11. Statistical methods

Statistical analysis was made using SAS System v9.4. All statistical decisions were performed considering two-sided tests and a significance level set at 0.05.

For quantitative variables, differences between groups were tested by means of t-tests or Mann–Whitney’s test when the normality assumption was not met. Data are expressed as mean ± SEM.

For qualitative variables, the association between variables was tested by means of the appropriate test (Chi-square test or Fisher’s exact when the Cochran’s rule was not satisfied).

## 3. Results

### 3.1. MSCs manufacturing

MSCs were compliant for all the quality controls performed: sterility, cell concentration, viability, morphology, potency test accumulative population doublings and mycoplasma contamination. The characterisation by flow cytometry was also compliant with the established specifications: positive markers were above 90% and negative markers below 3%.

### 3.2. Orthopaedic assessment

The screening visit determined that all the dogs included in the study had a normal orthopaedic evaluation: no joint effusion, no lameness, normal joint palpation, negative Drawer Sign and skin was intact in the injection point.

After the first administration no AE was reported in any group.

Only one AE was detected after the second administration, on day 29 per protocol, in one dog from the placebo group that presented effusion and mild lameness.

### 3.3. Clinical examination

Three dogs (1 EUC-MSC group and 2 placebos) had mildly increased temperature (~40°C) on days 15, 28 and 42, respectively. In all cases the animals were nervous and not presenting any other symptoms of disease. Therefore, it was considered as a casual finding, without being a sign of illness.

No sings of illness or abnormal findings occurred in any other dog during the trial in the clinical exams performed.

### 3.4. Laboratory examinations

No pathological alterations in blood test results were present in the laboratory analysis performed along the study.

Some causal findings (mildly increased haematocrit, haemoglobin levels, platelet count, MHC and HMCC values, glucose and AST levels) were observed equally in the three groups but they were not considered pathological given the population of dogs used during the study (high training police dogs), and considering the values found were not alarmingly out of range.

### 3.5. Glasgow scale evaluation

One of the dogs from the placebo group (same already mentioned in orthopaedic assessment), had a “Mobility” score of 1 on day 29. Also, a dog in the CAD-MSCs group was scored as 1 on day 31 (3 days after second administration).

All the other dogs and time points did not show abnormal scores.

### 3.6. Humoral response

**Figure 3:**
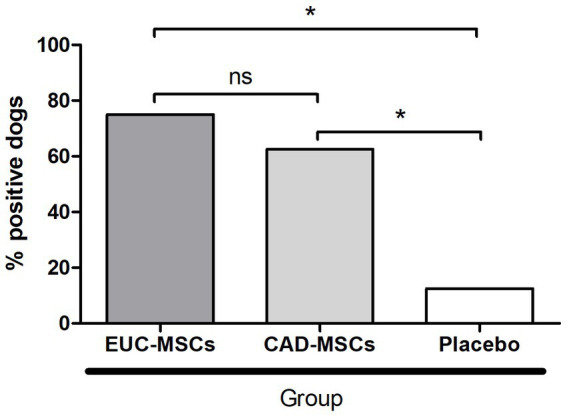
Percentage of dogs generating a humoral response after reinfiltration (day 42 per protocol) against the MSC type they received. Placebo sera were examined against both (EUC-MSCs and CAD-MSCs) and the result was negative for all of them against CAD-MSCs and one dog was positive against EUC-MSCs. *p < 0.05.

Before product administration none of the dogs showed antibodies against CAD-MSC nor EUC-MSCs. Fourteen days after second administration (day 42) 6/8 dogs (75%) EUC-MSC treated dogs and 5/8 dogs (63%) CAD-MSC treated dogs generated antibodies against EUC-MSC or CAD-MSC, respectively. One dog (13%) in the placebo group also showed a humoral response against EUC-MSCs but not against CAD-MSC (Figure 3).

All dogs showed antibodies against rabies (positive control) and 88% of them showed antibodies against BSA on day zero, before the treatment injection (Figure 3).

### 3.7. Cellular response

**Figure 4:**
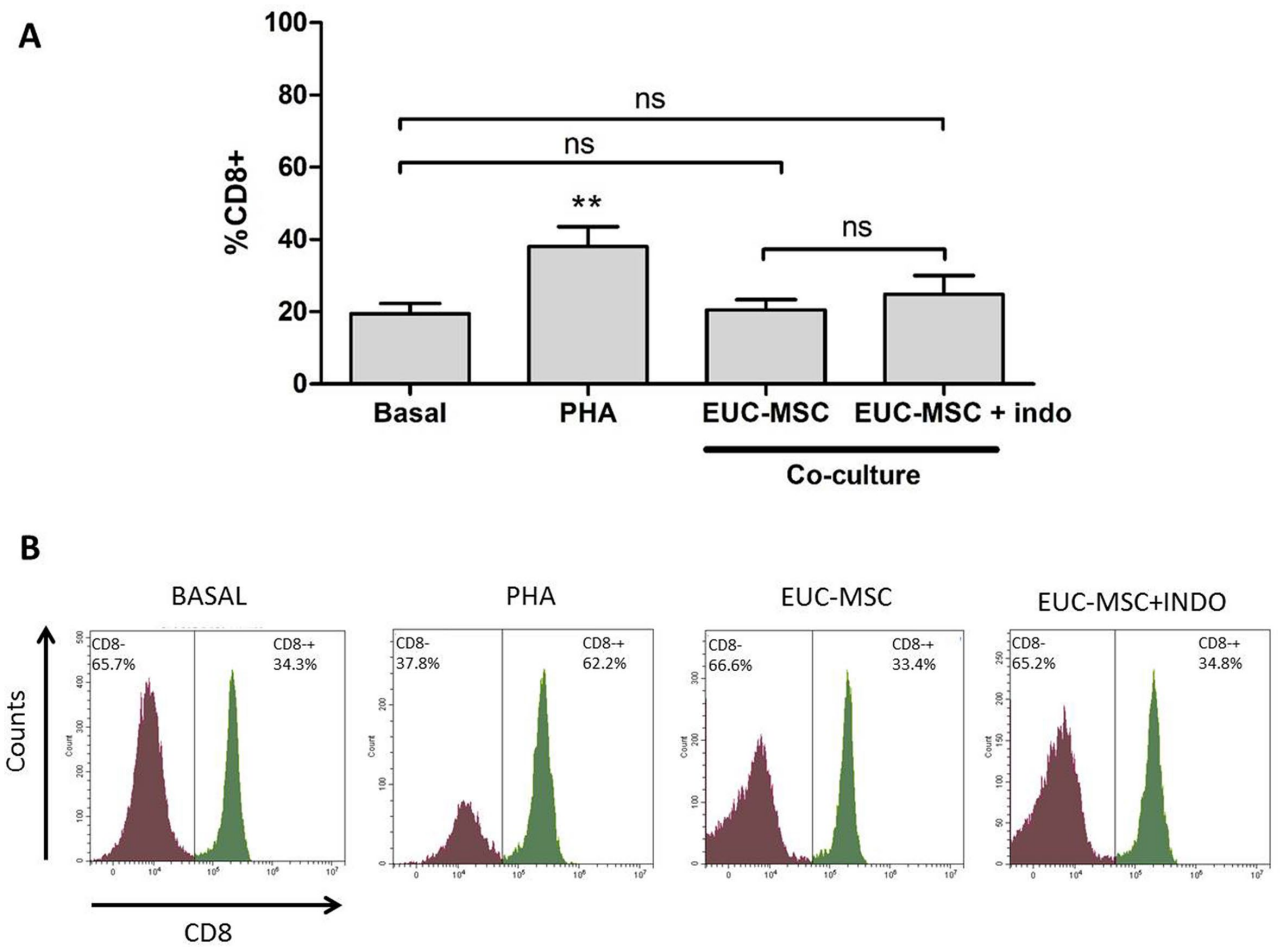
Percentage of memory lymphocytes CD8+ under each condition. **(A)** Mean percentage of memory lymphocytes CD8+ over the total lymphocyte population for each condition. Basal condition is compared with the rest of conditions. No differences were found in the co-culture compared neither to Basal nor between the two conditions of co-culture. **(B)** Example of an histogram of a donor under the different conditions. **p < 0.01 compared to Basal.

For EUC-MSC treated animals, basal PBMCs showed a mean percentage of lymphocytes CD8+ of 18.1% of the total lymphocyte population. In co-culture the mean % of CD8+ was 19.6% not being this difference statistically significant (Figure 4).

The co-culture with indomethacin did not show any difference with the one without it, demonstrating that MSCs are not inhibiting the activation of lymphocytes, but on the contrary, there was no activation.

**Figure 5:**
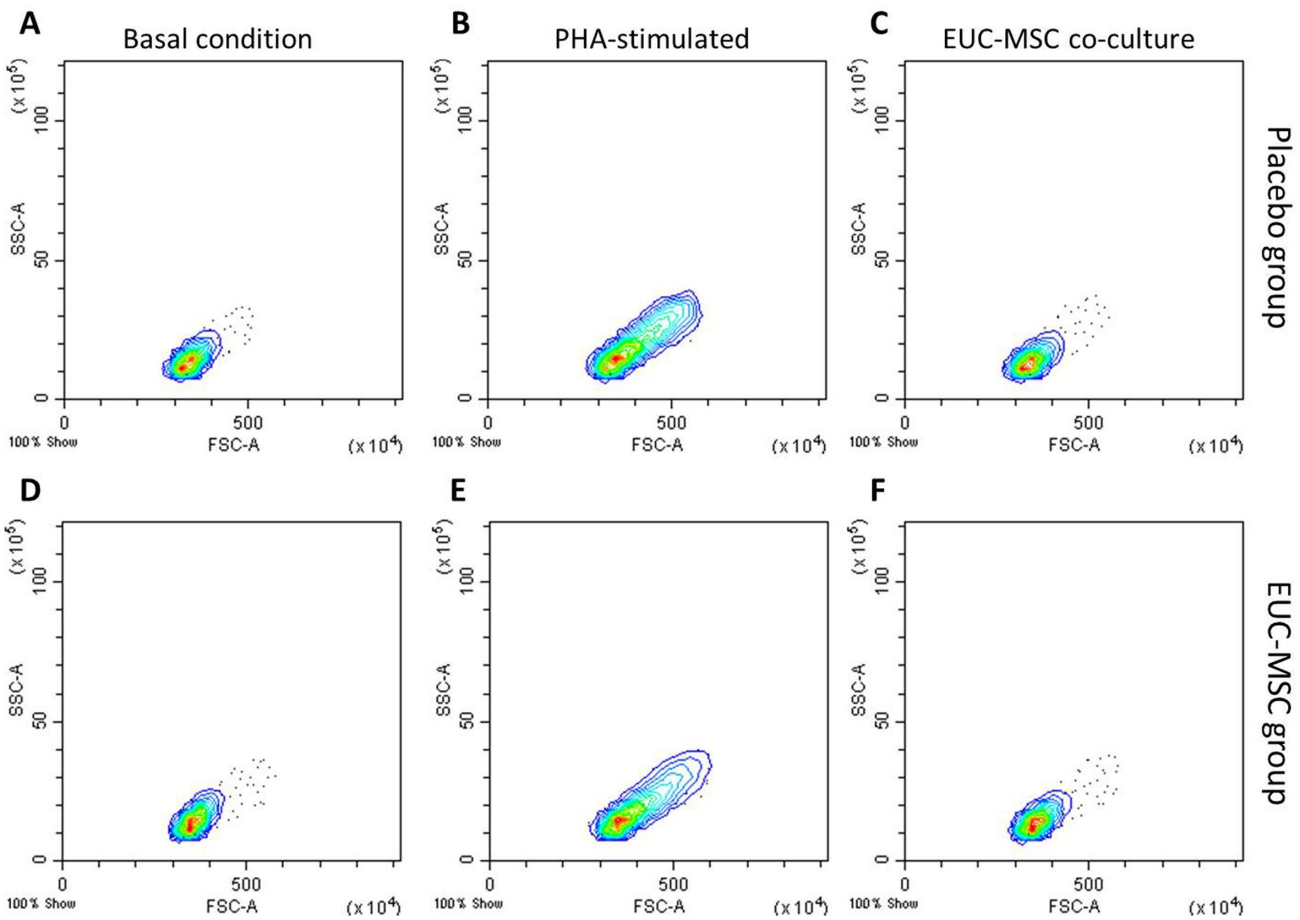
Flow cytometry analysis of lymphocyte populations from whole blood. **(A–C)** show basal condition, PHA-stimulated and co-cultured lymphocytes, respectively, from a placebo dog. **(D–F)** show the same from a EUC-MSCs treated dog.

In the lymphocyte population incubated with PHA the % of CD8+ was around 40% of the total lymphocyte population, confirming the ability of lymphocytes to be activated in the presence of a nonspecific stimulus. Activation of lymphocytes population is also observed by the increase in size and complexity which translates into a displacement of the population in the histogram upwards and to the right. This activation was not seen in the co-cultures (Figure 5).

In the placebo-treated dogs no differences were found compared with the EUC-MSCs treated dogs for any condition (data not shown).

MSCs showed a viability of more than 80% in all cases.

### 3.8. Adverse effects

Eleven non-product-related adverse events (AE) were detected in all groups indistinctly. These adverse events were: gastrointestinal symptoms, *Dirofilaria repens*, bite wound, epistasis and kennel cough. The AEs are reported individually in Table 3.

**Table 3:** Adverse events occurred during the study.

Dog ID	Group	Description of the adverse event	Duration (days)	Severity
ID1	Placebo	Kennel cough	7	Mild
ID2	EUC-MSCs	Vomited water during exploration	1	Mild
ID3	CAD-MSCs	Epistaxis during clinical exam	1	Mild
ID4	CAD-MSCs	Mild wound in the ear, bitten by another dog just before the first administration of the product	7	Mild
ID6	EUC-MSCs	Diarrhoea	3	Mild
ID7	EUC-MSCs	One vomit in the kennel with grass and bile aspect	1	Mild
ID8	Placebo	Diarrhoea	2	Mild
ID13	EUC-MSCs	*Dirofilaria repens* assintomatic is diagnosed by chance during the manipulation of the blood in the ficoll® during the cellular response test of day 0	5	Mild
ID17	Placebo	Diarrhoea	12	Mild
ID18	Placebo	Presented lameness grade 1 and effusion after the second administration	2	Mild
ID22	Placebo	Wound in right front limb, bitten by another dog	11	Moderate

The severity of the AE was assessed by the blinded veterinary surgeon.

In all the cases the adverse effects were considered to not be related to product administration since they were seen in same prevalence in all the groups. The most common AE detected was diarrhoea but clinical exploration was considered normal (temperature, hydration and abdominal palpation) in all the cases. These events were not considered pathologic.

## 4. Discussion

The aim of this study was to demonstrate the safety of the xenogeneic use of EUC-MSC in dogs. To prove our hypothesis the safety of EUC-MSCs when administered intra-articular in young healthy dogs in single and repeated administration was compared to single and repeated administration of allogeneic CAD-MSCs and placebo.

MSCs do not respond to conventional dose/response studies (44), so selecting the appropriated dose is often challenging in stem cells therapies. The dose 7.5 × 10^6^ cells/ml was calculated based on a multimodal approach. First, a comparison with the dose used in other species was done in order to determine the ideal dose comparing the joint size of the dog. For such comparison human and equine dose/size correlation was used (45). From this study it was determined that an intended dose for dogs should be between 5 and 10 million cell/joint. Then, a bibliographic research on the published intra-articular doses in dogs was done. According to the bibliography the most used dose for intra-articular administration in dogs is between 5 and 10 million (20, 21, 46–48). Last, the 7.5 × 10^6^ cells/ml dose was tested *in vivo* in natural injured dogs, in a pre-clinical study (data pending publication).

When making a safety study for a conventional treatment the procedure is to administer 2, 3 and 5-fold the effective dose of choice (49, 50). However, according to EMA guidelines overdose does not provide significant added value, when MSC-based product is administered locally (44, 48, 51).

The selected administration route was intra-articular. Intra-articular administration in dogs practice is not as common as in equine practice, so an intravenous approach would be an advantage in small animal practice (8). However, intravenous application of MSC is not without its drawbacks: (1) On the one hand, the dose required in intravenous treatments depends on the weight of the animal, so in large dogs the number of cells to inject will be much higher in IV administration than in intra-articular administration, which would presumably increase the cost of treatment. (2) In osteoarthritis, where there is a local inflammatory environment mediated by cytokines and inflammatory cells, applying MSCs locally is much more direct than IV, generating a more powerful anti-inflammatory/immunomodulatory effect. (3) Efficacy is greater in intra-articular applications. Reliably derived from the two previous points, less need for cells and direct local effect, in canine OA, the efficacy of IA MSCs is much higher than the efficacy of IV MSCs. Shah reported that the efficacy in the treatment of canine OA after IA application of MSC was 62% while after IV application the efficacy was <40%. Results from Shah and her group (52) after IA application of MSC in canine OA are in line with the ones reported by our group (28) where the efficacy of EUC-MSC in naturally occurring OA is greater than 65%.

In this study, the analysis of the treatment safety was thoroughly investigated at both local and systemic levels.

The dogs chosen for this study were a uniform population of police working dogs. They were young, healthy and very active dogs. Owners were asked to keep the dogs at rest on the day of infiltration, but the following day they continued with their usual training routine without showing any effect due to the administration. Only one dog in the placebo group showed effusion and mild lameness after the second administration. Since the AE occurred in a placebo-treated dog, it can be attributable to the intra-articular administration process rather than the product itself.

As for the potential immune response generated by the MSCs, it was measured at both humoral and cellular levels.

In the EUC-MSC group, 75% of the dogs developed antibodies against EUC-MSCs after repeated administration compared to 63% in the CAD-MSC group, nevertheless the specific antibody titers were not investigated in the present study, which has prevented a potential comparison between the titration between xenogenic and allogenic. In the present study, there was not harmful in any group, since the dogs did not show any symptom or abnormality in any of the examinations or laboratory tests. Furthermore, this difference is based on only one more dog responding to EUC-MSCs than to CAD-MSCs. The fact that the dogs treated with CAD-MSCs showed almost the same percentage of animals developing a serological response (only one dog less) as the dogs treated with EUC-MSCs, shows that it is not relevant that the cells are from a different species to the development or not of antibodies. The process of injecting these cells itself is prone to generate a minimal serological response, regardless of the origin of the cells. In all cases it is subclinical and does not cause adverse effects on the patient (15). In a recent review (53) the generation of donor specific antibodies was investigated in 555 patients, despite 12% of the patients developed antibodies the safety of MSC was proved in all the studies concluding that the generation of alloantibodies had no clinical relevance. Interestingly, antibodies against EUC-MSCs were also found in one dog (13%) in the placebo group, suggesting that dogs’ population could have exposure to xenogeneic horse-like tissue/cells/antigens in their normal life (maybe through vaccination or other exposures) or a cross-reactive response to the study.

In recent years, some authors claim that MSCs cannot be used for retreatment because they generate a cytotoxic response in the host (54, 55) that results in cell death. This makes the efficacy of the repeated treatments to be questioned. In these studies the cytotoxicity of MSCs on re-exposures is observed after an *in vitro* model mediated by the exogenous application of rabbit complement.

However, the reality after *in vivo* applications is that efficacy of MSC in allogeneic or xenogeneic administration is not reduced after repeated application. There is a strong amount of evidence that reported the efficacy of repeated administration of allogeneic MSC (56, 57) but also xenogeneic (58–60).

The reasons for the differences in the results between Berglund (54) and Rosa (55) *in vitro* model mediated by the addition of exogenous rabbit complement and the results in clinical practice or the present study, are difficult to establish, however, the potent immunomodulatory effect of MSCs and their complex *in vivo* mechanism of action could explain the different results.

Although MSCs are not completely ignored by the immune system and it is clear that they are capable of triggering a humoral response, as seen in the present study they are not able to transform this humoral response in a CD8+ cytotoxic response.

In this regard, the cytotoxicity of EUC-MSCs elicited by dog PBMCs was investigated by Garcia-Pedraza (61). EUC-MSCs were co-cultured with PBMCs from dogs that had received single or repeated doses of EUC-MSCs or placebo and the cytotoxicity was evaluated by MTT (3-[4,5-dimethylthiazo1-2-y1]-2,5-diphenyltetrazolium bromide) at different times. No cytotoxic effect was seen at any time point after single and repeated treatments. These results are in line with those reported by Van Hecke and Deputhy (62, 63).

As it has been discussed, UC-MSCs are more suitable for allogeneic or xenogeneic use than cells from other sources where the immune response appears to be more enhanced (64). Whilst EUC-MSCs are not associated with cytotoxicity, a humoral response can be seen after allogeneic or xenogeneic treatments. The generation of antibodies against MSC is well described in the literature; however the safety after re-treatments is also well reported by other authors (62, 63) and in the present work.

The presence of xenogeneic antibodies is not something new; many authors have described the presence of antibodies against BSA in humans, horses and dogs (65, 66). The origin of these antibodies seems to be due the traces of BSA and foetal bovine serum (FBS) in vaccines and other medications. Specifically, Mogues (66) investigated the prevalence of antibodies against BSA in a healthy population (55%) and in a population with lung cancer (51%). Mogues also investigated the effect on these antibodies after BSA re-exposure due to cancer surgical resection. After re-exposure, 98% of the patients showed antibodies against BSA with an increase of titter of 200-fold compared to before of re-exposure. Nevertheless, no patient showed any adverse event or pathological effect after the re-exposure to BSA; moreover elevated levels of anti-BSA antibodies were not associated with any detectable clinical events in either the healthy blood donors or the cancer patients.

As in the case of the present study with anti-MSC antibodies, it has been seen that anti-BSA antibodies have not been related to secondary cellular responses in humans (67), which may explain the innocuousness of re-exposure seen by Mogues (66). Therefore, it could be considered that in the absence of a cellular response, the presence of xenogeneic antibodies is not associated with safety or efficacy problems in re-exposures.

In the present study the presence of antibodies against BSA was also investigated at time 0 in all the dogs. As mentioned before, 88% of the dogs showed levels of xenogeneic antibodies against BSA before product administration. This demonstrates that the dogs are able to generate xenogeneic antibodies to manufacturing components of vaccines without relation to a negative immune response or safety problem in future canine vaccinations or re-exposure to BSA through diet or other sources. It would have been interesting to measure the anti-BSA titter after the administration of MSCs that may also contain traces of FBS in their composition, in order to determine if the administration of cell therapies is associated with an increase in anti-BSA antibodies. However, since anti-BSA antibodies were not associated with any adverse effects, it was not considered essential for the development of the study.

As discussed, it is more relevant the ability to generate a cellular response than the presence of antibodies *per se*, therefore the cellular response of canine PBMCs was investigated in the present work. For this work, CD8+ lymphocyte proliferation was expressed as an increase in the levels of CD8+ cells in peripheral blood of treated animals. A response mediated by cytotoxic CD8+ lymphocytes, would not allow re-infiltration with EUC-MSCs, which would prevent repeating the treatment. However, this study demonstrates the absence of a cytotoxic memory response indicating that re-infiltration with xenogeneic EUC-MSCs is safe and that the effectiveness would not be diminished. This memory response was evaluated by exposing the PBMCs from the dogs of the treatment group to EUC-MSCs in a co-culture, and measuring the differences in percentage of CD8+ memory lymphocytes (68). The generation of a memory response by the dog immune system would have resulted in an augment of CD8+ lymphocytes percentage upon re-contact with EUC-MSCs (69). PBMCs collected on day 42 of the protocol were chosen because it was considered the worst case scenario in which the cellular response, if any, would be the highest (14 days after the second administration). The co-culture conditions were followed as previously described in the literature (36). No significant increase in % CD8+ was seen in EUC-MSCs treated animals due to the re-exposition in the co-culture this means that the total percentage of CD8+ lymphocyte did not change along the study, nevertheless the proliferation was not addressed by other methods therefore could not be completely ruled out that proliferation could occur without changing the percentage of CD8+. On the other hand, no activation of PBMCs was observed since cells do not change in size and complexity, a typical variation when these cells are activated as shown in Figure 5, where PBMCs are stimulated with PHA (70). As observed, this absence of cellular response did not change in presence of indomethacin, showing that in the co-culture MSCs are not immunomodulating the memory response, but that the memory response does not exist.

Regarding the adverse events recorded in this study, the diarrheic episodes detected in both treatments and placebo group was considered to be a consequence of stress, confinement and training. It is well known that diarrheic episodes are common in working dogs and in highly nervous and excitable dogs (71).

Finally, no local or systemic adverse event has been identified after single or repeated administration in any of the animals.

In the present study it has been demonstrated that the single and repeated intra-articular administration of EUC-MSC is as safe as CAD-MSCs in young healthy dogs. As the main limitation of this study, it is worth noting the absence of dogs with natural disease or the evaluation of the efficacy of both single and repeated doses. Nevertheless, the same group recently conducted an efficacy and safety study (28) in dogs with naturally occurring OA treated with 7.5×10^6^ EUC-MSC (DogStem®) demonstrating the efficacy and safety of equine umbilical MSC in dogs with OA.

Altogether, these findings support the safety of the xenogeneic use of EUC-MSCs in dogs.

## Data availability statement

The raw data supporting the conclusions of this article will be made available by the authors, without undue reservation excepting that information considered confidential.

## Ethics statement

The animal study was reviewed and approved by Spanish Medicines Agency (AEMPS). Written informed consent was obtained from the owners for the participation of their animals in this study.

## Author contributions

AP designed the study and performed the data analysis. EP wrote the manuscript and performed the data analysis. MR conducted the cellular response test and the flow cytometry. LP conducted the humoral study. MG-C manufactured according GMP the MSCs injected in the animals. All authors contributed to the article and approved the submitted version.

## Funding

This research was funded by EquiCord S.L.

## Conflict of interest

EP, MG-C, MR, and AP are employed by EquiCord S.L. EquiCord owns DogStem’s European Marketing Authorisation.

The remaining authors declare that the research was conducted in the absence of any commercial or financial relationships that could be construed as a potential conflict of interest.

## Publisher’s note

All claims expressed in this article are solely those of the authors and do not necessarily represent those of their affiliated organizations, or those of the publisher, the editors and the reviewers. Any product that may be evaluated in this article, or claim that may be made by its manufacturer, is not guaranteed or endorsed by the publisher.
